# Low immunogenicity of whole virion, verocell-derived, inactivated, pandemic influenza H1N1 vaccine in HIV-infected patients

**DOI:** 10.1186/1758-2652-13-S4-P224

**Published:** 2010-11-08

**Authors:** H Lagler, K Grabmeier-Pfistershammer, V Touzeau-Römer, S Tobudic, A Rieger, H Burgmann

**Affiliations:** 1 Medical University of Vienna, Division of Infectious Diseases and Tropical Medicine, Department of Internal Medicine 1, Vienna, Austria; 2 Medical University of Vienna, Division of Immunology, Allergy and Infectious Diseases, Department of Dermatology, Vienna, Austria

## 

HIV infected individuals face an increased risk of serious illness from influenza. Current guidelines recommend vaccination of all HIV infected adults. Several studies have analysed the immunogenicity of adjuvant H1N1 vaccines in these individuals. However, no such analysis exists for Celvapan (Baxter), an inactivated H1N1 vaccine without adjuvants, derived from verocells and based on whole virions. Celvapan contains A/California/07/2009 (H1N1)v virus strain. Vaccination was provided in two i.m. doses, at least three weeks apart. This vaccine has been used widely in Austria during the recent H1N1 pandemic.

Adult HIV-1 infected individuals scheduled for H1N1 vaccination where included in this study. Serum samples were taken before the first vaccination (baseline) and after the second vaccination. Antibody titers were determined by hemagglutination inhibition (HAI) assay using chicken red blood cells and the Influenza A/H1N1pdm virus A/California/7/2009 (NIBSC 09/146). Clinical and HIV-related data were taken from patient charts. Seroconversion was defined as a ≥4-fold increase in antibody titer between the pre- and post vaccination serum, and a post-vaccination titer of ≥1:40 was considered protective. 78 patients were included in the study. 42 patients provided serum samples after the second vaccination. 74 % of the patients were male. Median age was 38.5 years. 21 patients had a VL below the limit of quantification (BLQ) since more than 12 months, 27 patients received currently HAART therapy with a VL BLQ. The median CD4 cell count was 442 cell/mm^3^ and the median CD4 nadir 209 cell/mm^3^. 38 % of the patients seroconverted after receiving both doses of the vaccine. Only 7 patients had HAI titers <1:40 before vaccination; all of them showed post-vaccination titers of >1:40. There was no significant difference with regard to age, the recent CD4 cell count or the CD4 nadir between seroconverters and non-responders and the share of patients with a recent VL BLQ or a VL BLQ for at least 12 months was roughly equal in both groups.

Seroconversion rates in HIV-infected individuals were significantly lower compared to those in otherwise healthy individuals in spite of a high percentage of individuals with well-controlled VL BLQ and high CD4 cell counts. In addition, seroconversion rate for HIV-infected individuals in this observational trial during 2009 pandemic flu season were lower than those recently published in studies using adjuvant H1N1 vaccines.

